# *Rogdi* Defines GABAergic Control of a Wake-promoting Dopaminergic Pathway to Sustain Sleep in *Drosophila*

**DOI:** 10.1038/s41598-017-11941-3

**Published:** 2017-09-12

**Authors:** Minjong Kim, Donghoon Jang, Eunseok Yoo, Yangkyun Oh, Jun Young Sonn, Jongbin Lee, Yoonhee Ki, Hyo Jin Son, Onyou Hwang, Changwook Lee, Chunghun Lim, Joonho Choe

**Affiliations:** 1 School of Life Sciences, Ulsan National Institute of Science and Technology, Ulsan, 44919 Republic of Korea; 2 Department of Biological Sciences, Korea Advanced Institute of Science and Technology, Daejeon, 34141 Republic of Korea; 3 Cell Logistics Research Center, Gwangju Institute of Science and Technology, Gwangju, 61005 Republic of Korea; 4 Department of Biochemistry and Molecular Biology, University of Ulsan College of Medicine, Seoul, 05505 Republic of Korea

## Abstract

Kohlschutter-Tönz syndrome (KTS) is a rare genetic disorder with neurological dysfunctions including seizure and intellectual impairment. Mutations at the *Rogdi* locus have been linked to development of KTS, yet the underlying mechanisms remain elusive. Here we demonstrate that a *Drosophila* homolog of *Rogdi* acts as a novel sleep-promoting factor by supporting a specific subset of gamma-aminobutyric acid (GABA) transmission. *Rogdi* mutant flies displayed insomnia-like behaviors accompanied by sleep fragmentation and delay in sleep initiation. The sleep suppression phenotypes were rescued by sustaining GABAergic transmission primarily via metabotropic GABA receptors or by blocking wake-promoting dopaminergic pathways. Transgenic rescue further mapped GABAergic neurons as a cell-autonomous locus important for *Rogdi*-dependent sleep, implying metabotropic GABA transmission upstream of the dopaminergic inhibition of sleep. Consistently, an agonist specific to metabotropic but not ionotropic GABA receptors titrated the wake-promoting effects of dopaminergic neuron excitation. Taken together, these data provide the first genetic evidence that implicates *Rogdi* in sleep regulation via GABAergic control of dopaminergic signaling. Given the strong relevance of GABA to epilepsy, we propose that similar mechanisms might underlie the neural pathogenesis of *Rogdi*-associated KTS.

## Introduction

Neurological disorders caused by single-gene mutations are important genetic models to understand how individual genes execute their roles to support the development and function of the brain, as in the case of Kohlschutter-Tönz syndrome (KTS). KTS patients display developmental delays and psychomotor regression^1^. The most prominent symptoms include amelogenesis imperfecta, early-onset seizures, and intellectual disabilities. Linkage analyses followed by genomic sequencing have revealed that most, if not all, KTS patients have homozygous nonsense, frameshift deletion, or splicing site mutations at the *Rogdi* locus^2–6^. ROGDI protein expression is not detectable in affected individuals^2^, indicating that the loss of *Rogdi* function is responsible for the pathogenesis of KTS. In wild-type human tissues, *Rogdi* transcripts are ubiquitously expressed while the highest enrichment is evident in adult brain and spinal cord^2, 3^. This observation is consistent with the neurological phenotypes observed in KTS patients.

Given that *Rogdi* homologs are relatively well conserved in higher eukaryotes, animal models may facilitate our understanding of *Rogdi*-dependent neural processes. In fact, *Rogdi* was initially identified in a *Drosophila* genetic screen as a memory-relevant gene and was thus named after one of Pavlov’s dogs^7^. Sequence analyses of *Rogdi* homologs revealed a putative leucine zipper (ZIP) motif, which could mediate the dimerization of DNA-binding basic ZIP (bZIP) transcription factors^5^. Interestingly, ROGDI proteins localize to the nuclear envelope in cultured human cells^2^, although they lack basic amino acid residues that are typically located at the N-terminus of the ZIP domain and are required for the DNA-binding and nuclear localization of the bZIP transcription factors^8^. Nonetheless, few or no studies have demonstrated the biological activity of *Rogdi* ^9^ and genetic models for *Rogdi* homologs have not been reported yet. Therefore, how *Rogdi* exerts its physiological roles particularly in the central nervous system and how its mutation leads to the development of KTS are largely unknown.

In the course of our genetic studies to elucidate genes and regulatory pathways involved in sleep behaviors, we identified novel sleep mutant alleles in the *Drosophila Rogdi* gene. Here, we employed the sleep-promoting effects of *Rogdi* as a readout of its neural function and demonstrated that *Rogdi* acts cell-autonomously in GABAergic neurons to enhance metabotropic GABA transmission and thereby sustain sleep. In addition, dopaminergic rescue of *Rogdi* mutant sleep revealed a novel sleep-regulatory mechanism that functionally links a specific subset of sleep-promoting GABAergic neurons to a wake-promoting dopaminergic pathway. Since epilepsy, a well penetrated phenotype in KTS patients, implicates GABAergic transmission^10–12^ and sleep disorders^13–16^, our findings provide an important genetic clue to understanding the molecular and neural pathogenesis of KTS.

## Results

### Loss-of-function Mutations in *Rogdi* Suppress Sleep Behaviors in *Drosophila*

**Figure 1:**
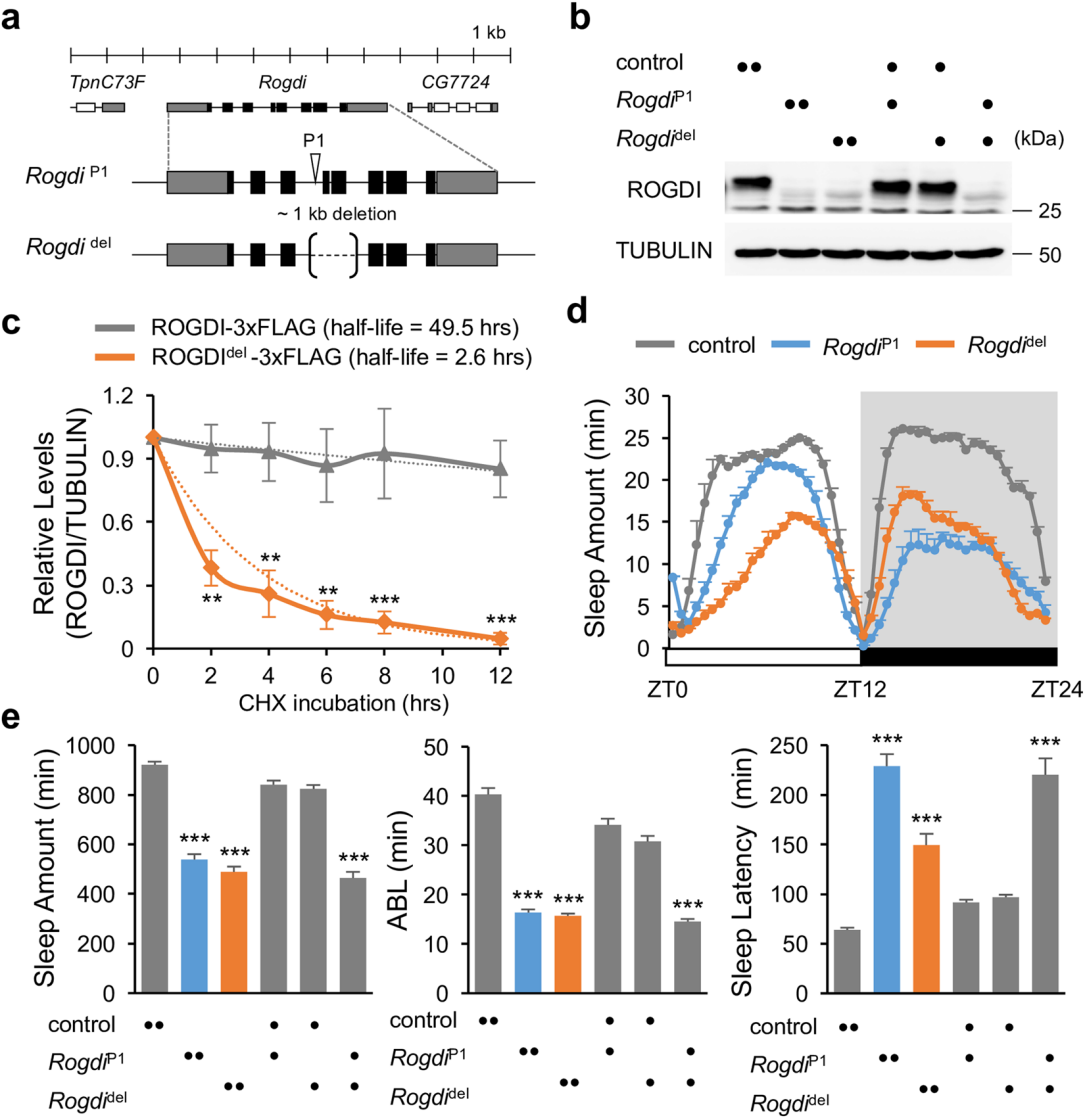
*Rogdi* mutation suppresses sleep in *Drosophila*. (**a**) A schematic of *Rogdi* mutant alleles. P element insertion (*Rogdi* ^P1^) and genomic deletion (*Rogdi* ^del^) are depicted in the context of the *Rogdi* locus. (**b**) Fly head extracts from wild-type and *Rogdi* mutants were immunoblotted with anti-ROGDI (top) and anti-TUBULIN (bottom, loading control) antibodies. Images were cropped from full-length blots shown in Supplementary Fig. 1. Protein size markers were shown on the right. (**c**) Wild-type ROGDI or ROGDI^del^ proteins with a 3xFLAG epitope tag were transiently expressed in *Drosophila* S2 cells and their decay rate (i.e., half-life) was measured after cycloheximide (CHX)-induced blockade of protein synthesis. Data represent average ± SEM (n = 3). Two-way ANOVA detected significant effects of genotypes (F[1,24] = 82.58, *P* < 0.0001). **P < 0.01, ***P < 0.001 to wild-type at each time-point as determined by Bonferroni post hoc test. (**d**) Daily sleep profiles of control and *Rogdi* mutant flies in 30 minute intervals. Sleep behaviors in individual flies were quantitatively analyzed under 12-hour light (white bar): 12-hour dark (black bar) (LD) cycles at 25 °C. Data represent average ± SEM (n = 61–126). ZT, zeitgeber time (lights-on at ZT0; lights-off at ZT12). (**e**) Daily sleep amount, average sleep bout length (ABL), and sleep latency in homozygous and trans-heterozygous *Rogdi* mutants were compared with those in wild-type and heterozygous controls. ***P < 0.001 to wild-type and heterozygous controls as determined by one-way ANOVA, Tukey post hoc test.

Sleep is physiologically essential for animals. Genetic models for sleep and sleep-related disorders have been established in multiple species in order to understand why we sleep and how sleep homeostasis occurs^17–19^. In addition, sleep-relevant genes have been successfully established in *Drosophila* sleep models by isolating novel sleep mutants and characterizing their sleep-modulatory effects^20–25^. We took a similar approach to identify a transgenic fly that harbors a genomic insertion of a transposable P element and displays insomnia-like sleep behaviors in 12-hour light: 12-hour dark (LD) cycles (Fig. 1). Sequence mapping of the transgene revealed insertion of the P element in the third intron at the *Rogdi* locus (*Rogdi* ^P1^) (Fig. 1a). We further excised the *Rogdi* ^P1^ insertion to generate a ~1 kb genomic deletion allele (*Rogdi* ^del^) that caused an in-frame deletion of amino acid residues 16–83 in the gene product. Mutant flies homozygous for either the *Rogdi* insertion or the deletion allele barely expressed ROGDI proteins in head extracts as assessed by immunoblotting with polyclonal anti-ROGDI antibody (Fig. 1b, Supplementary Fig. 1). To determine if the in-frame deletion caused by *Rogdi* ^del^ allele could give rise to mutant ROGDI proteins, we overexpressed the cDNAs corresponding to either wild-type or *Rogdi* ^del^ allele in *Drosophila* S2 cells and examined their protein products using an epitope-tag. Indeed, wild-type and ROGDI^del^ proteins were comparably detectable in immunoblots of total cell extracts but the mutant ROGDI^del^ proteins had much shorter half-life (~2.6 hours) than wild-type (~49.5 hours) (Fig. 1c, Supplementary Fig. 2). Moreover, ROGDI^del^ proteins were localized to distinct, cytoplasmic inclusions (Supplementary Fig. 3a). This subcellular localization contrasted with that of wild-type ROGDI proteins which were evenly distributed in both nucleus and cytoplasm of S2 cells or adult fly neurons (Supplementary Fig. 3b). Collectively, these data indicate that the *Rogdi* ^del^ allele substantially affects the proteostasis of ROGDI proteins in general (See Discussion).

Importantly, the daily amount of sleep was substantially decreased in flies homozygous or trans-heterozygous for the mutant *Rogdi* alleles (Fig. 1d,e). The two mutant alleles led to distinct sleep profiles, likely due to the difference in their allelic nature. The sleep suppression phenotype was accompanied by sleep fragmentation: the average sleep bout length (ABL) was shorter in *Rogdi* mutants than in control flies (Fig. 1e). In addition, *Rogdi* mutants took longer to initiate their first sleep bout after lights off when looking at the sleep latency. Sleep fragmentation was also evident in female flies homozygous for the *Rogdi* deletion (Supplementary Fig. 4). However, *Rogdi* mutant phenotypes regarding total sleep amount and sleep latency were observed in virgins but not in mated females, possibly indicating that reproduction and/or neural processes relevant to post-mating responses might modulate *Rogdi* effects on sleep in female flies^26, 27^. Nonetheless, these data suggest that *Rogdi* is a novel sleep-promoting gene important for both sleep initiation and maintenance.

### Insomnia-like Behaviors in *Rogdi* Mutants Do Not Implicate Deficits in Circadian Rhythms or Sleep Homeostasis

**Figure 2:**
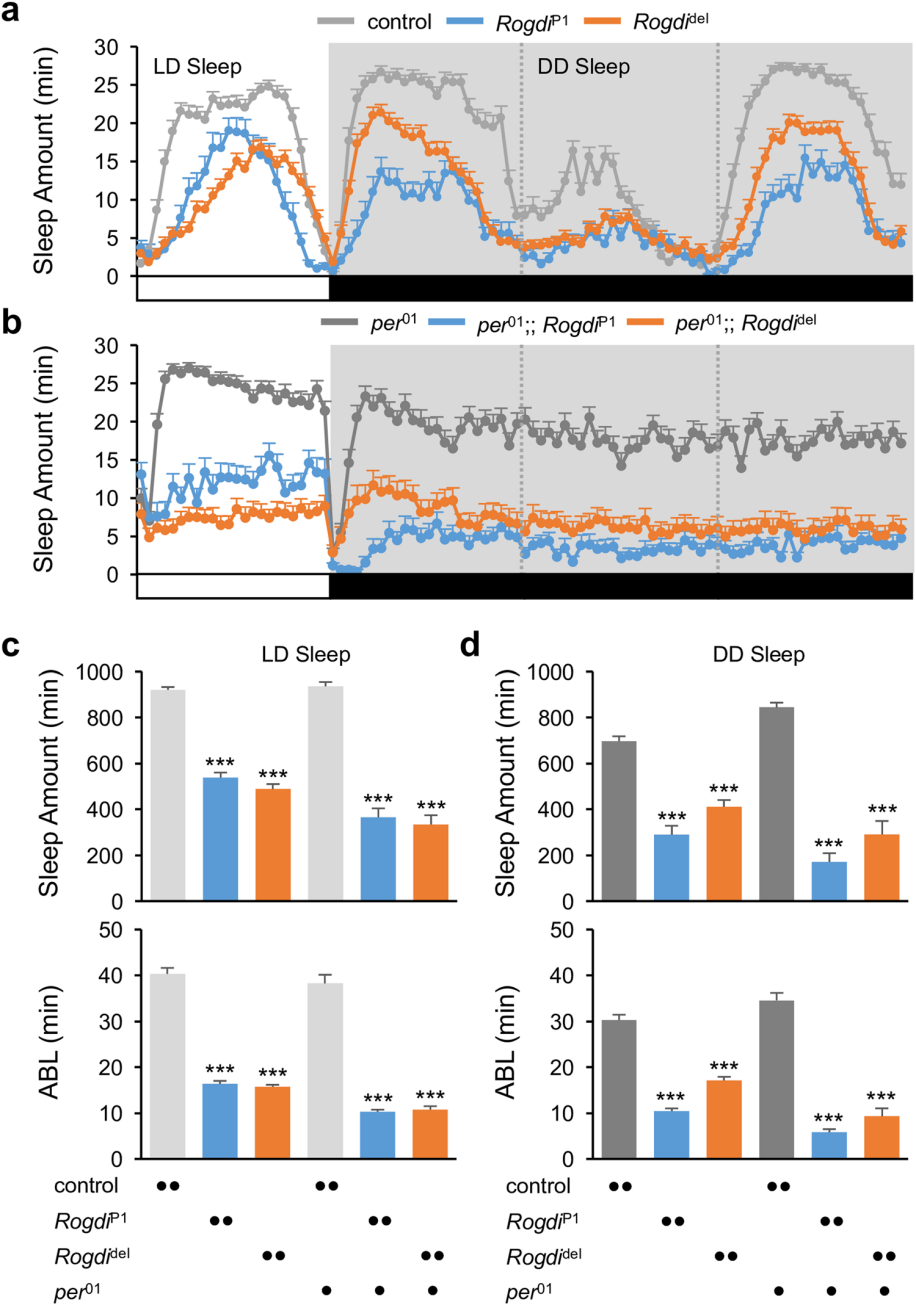
Wake-promoting effects of *Rogdi* mutation require neither LD cycles nor the functionality of circadian rhythms. (**a**,**b**) Daily sleep profiles of control and *Rogdi* mutant flies in wild-type (**a**) or clock-less *per* ^01^ mutant backgrounds (**b**). Sleep behaviors in individual flies were monitored in LD cycles followed by constant dark (DD) cycles at 25 °C. Data represent average ± SEM (n = 35–126). (**c**,**d**) Daily sleep amount (top) and average sleep bout length (ABL, bottom) in LD (**c**) or the first DD (**d**) cycles were compared among control and *Rogdi* mutant flies in wild-type or *per* ^01^ mutant backgrounds. ***P < 0.001 to control or *per* ^01^ mutants as determined by one-way ANOVA, Tukey post hoc test.

Circadian rhythms and sleep homeostasis have long been considered as two essential processes that shape sleep behaviors^28^. To understand how these two factors are implicated in *Rogdi*-dependent sleep, we examined *Rogdi* mutant sleep in different environmental or genetic conditions. The sleep suppression by *Rogdi* mutation persisted either in constant darkness or in a genetic background that harbored a hemizygous loss-of-function mutant allele of the circadian clock gene *period* ^29, 30^ (Fig. 2). Since the clock-less *per* mutants do not display daily oscillations in clock gene expression and locomotor behaviors, these data imply that *Rogdi* effects on sleep require neither LD cycles nor the functionality of circadian rhythms.

**Figure 3:**
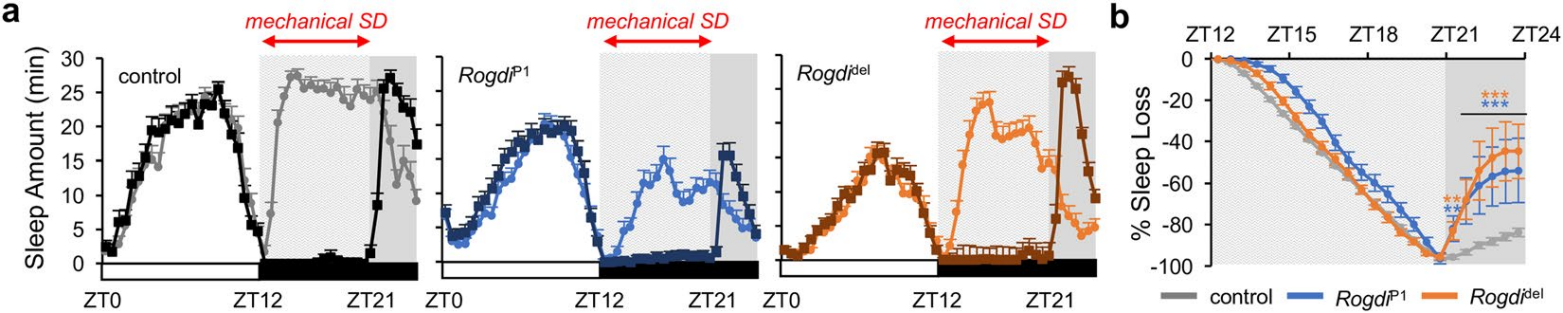
*Rogdi* mutation does not dampen sleep rebound after mechanical sleep deprivation. (**a**) Sleep behaviors in individual flies were monitored in LD cycles at 25 °C. Where indicated, flies were subject to the mechanical stimulus for the first 9 hours in the D phase (from ZT12 to ZT21) and their sleep rebound was quantitatively analyzed for the last 3 hours in the D phase (from ZT21 to ZT24). Averaged sleep profiles on the day of mechanical sleep deprivation (SD) (darker lines) were overlaid by those on the previous day (lighter lines). ZT, zeitgeber time (lights-on at ZT0; lights-off at ZT12). (**b**) Cumulative sleep loss was calculated in individual flies and averaged per each genotype. To calculate %sleep loss at each time-point, total sleep amount from ZT12 to a given time-point on the day of SD was subtracted by that on the previous day and then normalized to the total sleep amount from ZT12 and ZT21 on the previous day. Data represent average ± SEM (n = 31–40). Two-way repeated-measures ANOVA detected significant effects of genotypes (F[2,100] = 4.707, *P* = 0.0111) and their interaction with time-points (F[46,2300] = 2.495, *P* < 0.0001). **P < 0.01, ***P < 0.001 to control at each time-point as determined by Dunnett post hoc test.

To examine whether *Rogdi* contributes to sleep homeostasis, we employed a mechanical sleep-deprivation protocol^31^. We optimized the visibility of homeostatic sleep regulation by applying 9 hours (rather than overnight) of mechanical stimulus starting from lights-off in the LD cycle. Sleep rebound was then measured for the last 3 hours in the D phase so that lights-on would not mask the compensatory increase in sleep drive after sleep deprivation. Under these conditions, *Rogdi* mutants displayed higher sleep rebound than control flies as assessed by the percentage of sleep gain during the recovery period (Fig. 3). While we do not exclude the possibility that longer baseline sleep in control flies and possible ceiling effects could have underestimated their relative sleep rebound, these data imply that *Rogdi* suppresses homeostatic sleep gain after sleep loss. In fact, this phenotype contrasts with sleep-promoting effects of *Rogdi* on baseline sleep but is consistent with a previous observation that sleep-regulatory pathways for baseline and recovery sleep could be genetically separable (See Discussion)^32^.

### A Wake-promoting Dopaminergic Pathway Mediates *Rogdi* Effects on Sleep

**Figure 4:**
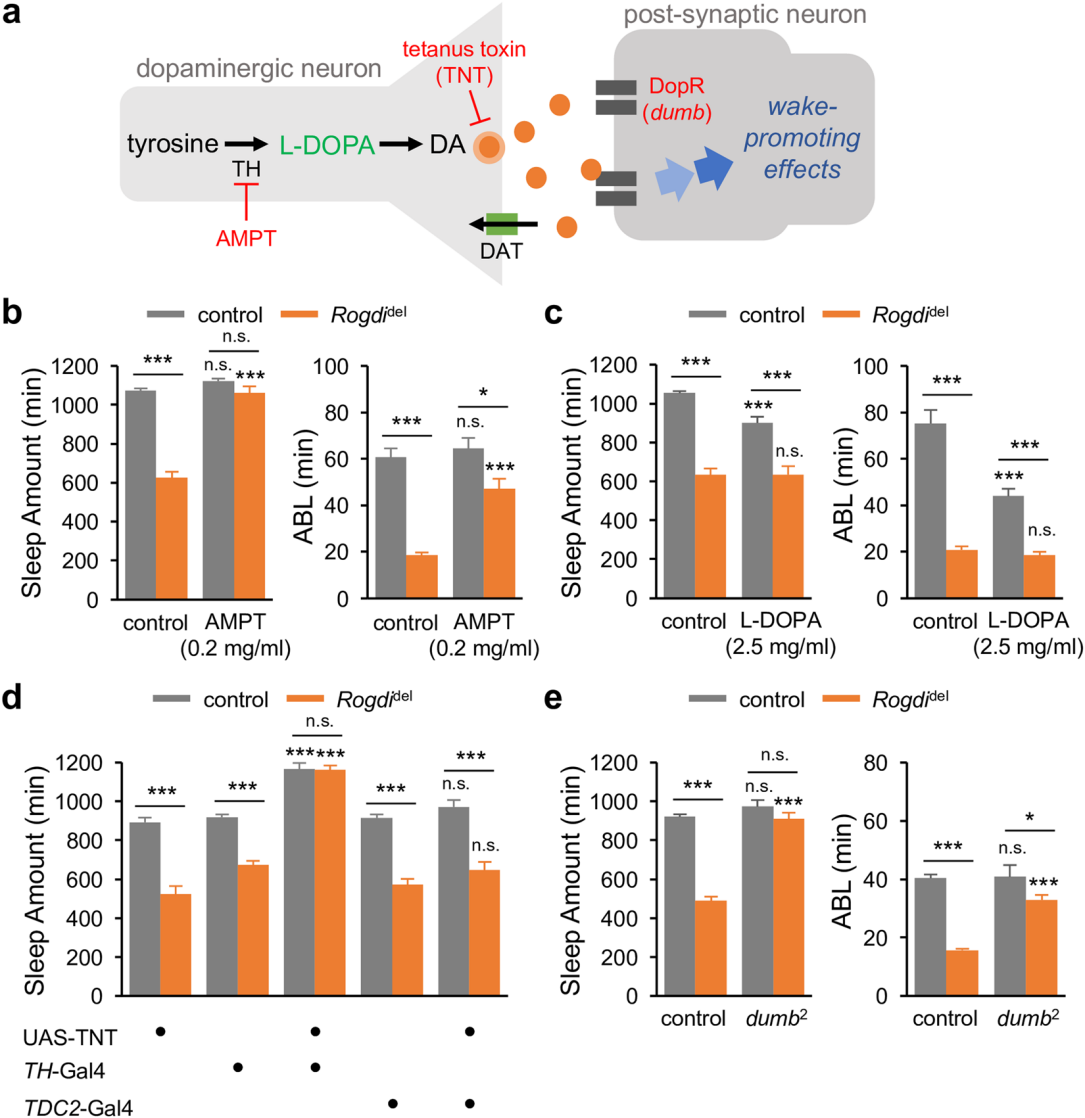
A dopaminergic pathway mediates the wake-promoting effects of *Rogdi* mutation. (**a**) A schematic of a dopaminergic synapse. AMPT, alpha-methyl-p-tyrosine (an inhibitor of tyrosine hydroxylase); DA, dopamine; DAT, dopamine transporter; DopR, D1-like dopamine receptor; L-DOPA, L-3,4-dihydroxyphenylalanine (a precursor of DA); TH, tyrosine hydroxylase. (**b**) Oral administration of AMPT rescues short baseline sleep in *Rogdi* mutants. Sleep behaviors in individual flies were analyzed similarly to the data presented in Fig. 1. Gray and orange bars indicate wild-type (*w* ^*1118*^) and *Rogdi* ^del^ mutant backgrounds, respectively. Data represent average ± SEM (n = 30–63). Two-way ANOVA detected significant interaction between *Rogdi* mutation and AMPT effects on sleep amount (F[1,155] = 71.13, *P* < 0.0001) and average sleep bout length (ABL) (F[1,155] = 9.102, *P* = 0.0030). (**c**) *Rogdi* mutation desensitizes the wake-promoting effects of L-DOPA (n = 34–63). Two-way ANOVA detected significant interaction between *Rogdi* mutation and L-DOPA effects on sleep amount (F[1,185] = 8.59, *P* = 0.0038) and ABL (F[1,185] = 10.2, *P* = 0.0017). (**d**) Blocking synaptic transmission in dopaminergic (*TH* > TNT, tetanus toxin light chain) but not octopaminergic (*TDC2* > TNT) neurons masks the wake-promoting effects of *Rogdi* mutation (n = 23–80). Two-way ANOVA detected significant interaction of *Rogdi* mutation with *TH* > TNT (F[2,230] = 15.42, *P* < 0.0001) but not with *TDC2* > TNT (F[2,191] = 0.1922, *P* = 0.8253). (**e**) Hypomorphic mutation in a dopamine receptor gene, *dumb*, suppresses the short sleep phenotypes in *Rogdi* mutants (n = 30–126). Two-way ANOVA detected significant genetic interaction between *Rogdi* and *dumb* mutations on sleep amount (F[1,280] = 44.72, *P* < 0.0001) and ABL (F[1,280] = 28.05, *P* < 0.0001). n.s., not significant, *P < 0.05, ***P < 0.001 to no-drug or heterozygous controls in the same genetic backgrounds as determined by Tukey post hoc test.

To elucidate the neural basis underlying the wake-promoting effects of *Rogdi* mutation, we first examined whether *Rogdi* mutants could be rescued by pharmacological treatment targeting specific neurotransmitter pathways. Tyrosine hydroxylase (TH) is a rate-limiting enzyme for the conversion of L-tyrosine to L-3,4-dihydroxyphenylalanine (L-DOPA), a precursor of the neurotransmitter, dopamine (DA) (Fig. 4a). Dopaminergic signaling pathways have been well established as having wake-promoting effects in both *Drosophila* and mammals^21, 33, 34^. In particular, a subset of TH-expressing dopaminergic neurons and their downstream postsynaptic target (D1-like DA receptor-expressing cells in the dorsal fan-shaped body) constitute a neural circuit that is important for promoting wakefulness in *Drosophila* ^35–37^. Importantly, we found that oral administration of a sub-dose of the TH inhibitor alpha-methyl-p-tyrosine (AMPT) fully rescued the total sleep amount in *Rogdi* mutants (Fig. 4b). AMPT also significantly lengthened ABL in *Rogdi* mutants but not in control flies, indicating that AMPT treatment substantially restored sleep consolidation in *Rogdi* mutants. Conversely, L-DOPA administration shortened the total sleep amount and ABL in control flies but not in *Rogdi* mutants (Fig. 4c), indicating that *Rogdi* mutants are more resistant to the wake-promoting effects of L-DOPA. At the dosages used in our experiments, both AMPT and L-DOPA affected sleep latency comparably in control flies and *Rogdi* mutants (Supplementary Fig. 5).

To further validate the implication of dopaminergic transmission in *Rogdi*-dependent sleep regulation, we examined if genetic disruption in the DA pathway could mask *Rogdi* effects on baseline sleep. Transgenic expression of tetanus toxin light chain (TNT) in TH-expressing neurons could block neurotransmission specifically at the dopaminergic synapses. This genetic manipulation indeed suppressed the wake-promoting effects of *Rogdi* mutation (Fig. 4d, Supplementary Fig. 6). By contrast, synaptic blockade of the wake-promoting octopaminergic transmission^24^ did not affect the short sleep phenotypes in *Rogdi* mutants, verifying the specific involvement of the dopaminergic circuit in *Rogdi*-dependent sleep. In addition, hypomorphic mutation in the D1-like DA receptor *dumb* ^35, 36^ also rescued *Rogdi* mutant phenotypes (Fig. 4e, Supplementary Fig. 7). Collectively, our pharmacological and genetic data demonstrate that the wake-promoting DA pathway mediates the sleep suppression observed in *Rogdi* mutants.

### GABAergic Transmission via Metabotropic GABA Receptors Supports *Rogdi*-dependent Sleep

**Figure 5:**
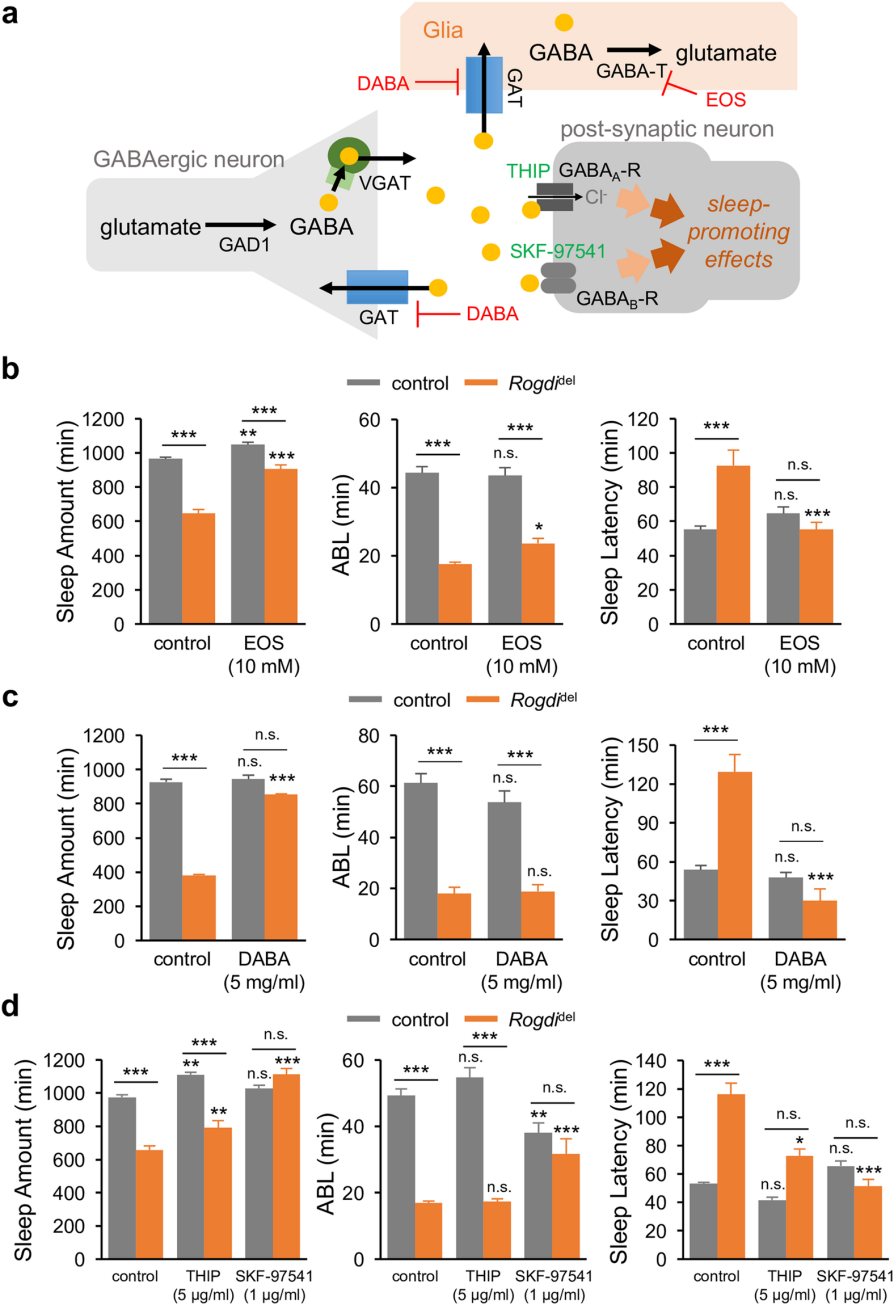
*Rogdi*-dependent sleep involves a sleep-promoting pathway of GABAergic transmission via metabotropic GABA receptors. (**a**) A schematic of GABAergic synaptic transmission. DABA, DL-2,4-diaminobutyric acid (GAT inhibitor); EOS, ethanolamine O-sulfate (GABA-T inhibitor); GABA_A_-R, ionotropic GABA receptor; GABA_B_-R, metabotropic GABA receptor; GABA-T, GABA transaminase; GAD1, glutamate decarboxylase 1; GAT, GABA transporter; SKF-97541, 3-Aminopropyl(methyl)phosphinic acid (GABA_B_-R agonist); THIP, 4,5,6,7-tetrahydroisoxazolo[5,4-c]pyridin-3-ol (GABA_A_-R agonist); VGAT, vesicular GABA transporter. (**b,c**) Oral administration of EOS (**b**) or DABA (**c**) rescues sleep quantity and sleep latency in *Rogdi* mutants. Sleep behaviors in individual flies were analyzed similarly to the data presented in Fig. 1. Gray and orange bars indicate wild-type (*w* ^*1118*^) and *Rogdi* ^del^ mutant backgrounds, respectively. Data represent average ± SEM (n = 108–120 for EOS; n = 36–78 for DABA). Two-way ANOVA detected significant interaction of *Rogdi* mutation with EOS and DABA effects on sleep amount (F[1,449] = 21.43, *P* < 0.0001 for EOS; F[1,209] = 55.2, *P* < 0.0001 for DABA), average sleep bout length (ABL) (F[1,449] = 4.284, *P* = 0.0390 for EOS only) and sleep latency (F[1,449] = 18.61, *P* < 0.0001 for EOS; F[1,209] = 16.61, *P* < 0.0001 for DABA). (**d**) An agonist of GABA_B_-R (SKF-97541) but not GABA_A_-R (THIP) fully rescues the short sleep phenotypes in *Rogdi* mutants (n = 29–92). Two-way ANOVA detected significant interaction of *Rogdi* mutation with SKF-97541 effects on sleep amount (F[1,228] = 56.39, *P* < 0.0001), ABL (F[1,228] = 33.81, *P* < 0.0001) and sleep latency (F[1,228] = 14.38, *P* = 0.0002) but not with THIP. n.s., not significant, *P < 0.05, **P < 0.01, ***P < 0.001 to no-drug controls in the same genetic backgrounds as determined by Tukey post hoc test.

Gamma-aminobutyric acid (GABA) is known to be involved in sleep regulation and its sleep-promoting effects are well conserved between flies and mammals^17^. We thus assessed possible roles of the inhibitory neurotransmitter GABA in the wake-promoting effects of *Rogdi* mutation (Fig. 5a). Mitochondrial GABA transaminase (GABA-T) metabolizes GABA into succinic semialdehyde to suppress GABAergic transmission^38^. A low dose of the GABA-T inhibitor, ethanolamine O-sulfate (EOS) modestly elevated sleep amount in control flies, whereas it fully rescued sleep latency and substantially increased sleep amount in *Rogdi* mutants (Fig. 5b). Since GABA-T expressed in glial cells has been implicated in promoting wakefulness^38^, we next asked if elevated GABA levels in the synaptic clefts or glia could rescue *Rogdi* mutant sleep. Synaptic GABA levels are lowered by GABA transporter (GAT) that translocates extracellular GABA back to presynaptic neurons and glial cells, thereby restraining GABAergic transmission^39^. We found that the short sleep phenotypes in *Rogdi* mutants were also rescued by the GAT inhibitor, DL-2,4-diaminobutyric acid (DABA) (Fig. 5c). These data support that the pharmacological elevation of GABAergic transmission could mask the wake-promoting effects of *Rogdi* mutation, suggesting that deficits in the sleep-promoting GABAergic pathway might be responsible for the sleep phenotypes in *Rogdi* mutants.

To independently validate this hypothesis, we examined the sleep-promoting effects of GABA receptor agonists^40, 41^. Oral administration of 4,5,6,7-tetrahydroisoxazolo[5,4-c]pyridin-3-ol (THIP), an agonist of ionotropic GABA receptors, promoted sleep quantity comparably in control and *Rogdi* mutant flies while it shortened sleep latency in *Rogdi* mutants (Fig. 5d). By contrast, a low dose of the metabotropic GABA receptor agonist SKF-97541 affected ABL very modestly in control flies while it fully restored normal sleep behaviors in *Rogdi* mutants. These data suggest that sleep-promoting GABAergic transmission via metabotropic GABA receptors might be prominently compromised by *Rogdi* mutations. Taken together, our pharmacological and genetic evidence demonstrates that *Rogdi*-dependent sleep regulation involves two opposing effects of the wake-promoting DA and sleep-promoting GABA pathways.

### *Rogdi* in GABAergic Neurons Acts Upstream of a Wake-promoting Dopaminergic Pathway to Promote Sleep

**Figure 6:**
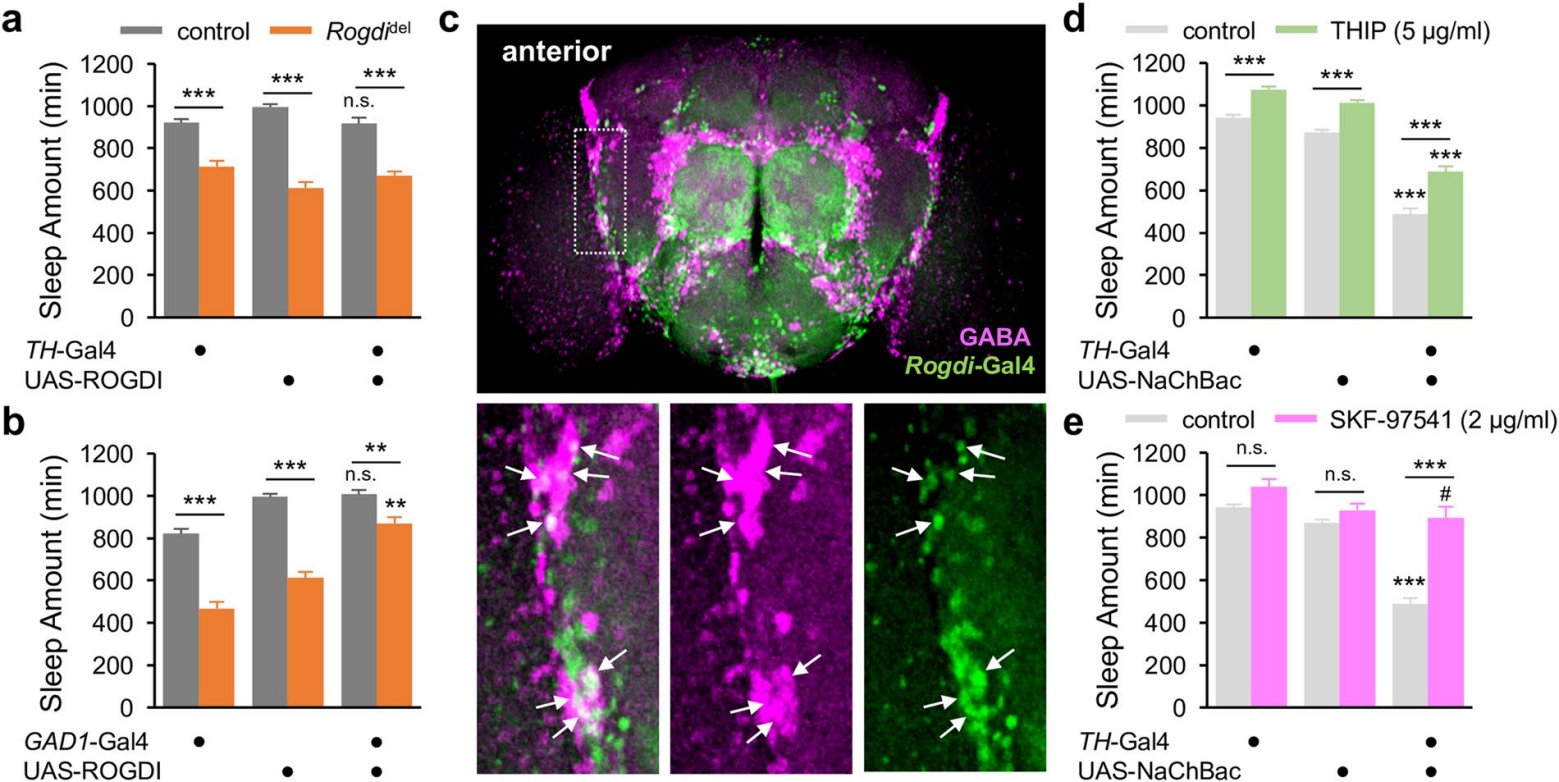
*Rogdi* in GABAergic neurons promotes sleep. (**a**,**b**) Transgenic ROGDI expression in GABAergic (*GAD1* > ROGDI) but not dopaminergic (*TH* > ROGDI) neurons rescues short sleep in *Rogdi* mutants. Sleep behaviors in individual flies were analyzed similarly to the data presented in Fig. 1. Gray and orange bars indicate wild-type (*w* ^*1118*^) and *Rogdi* ^del^ mutant backgrounds, respectively. Data represent average ± SEM (n = 24–43). In the dopaminergic rescue, two-way ANOVA detected significant effects of *Rogdi* mutation (F[1,229] = 220.3, *P* < 0.0001) but not *TH* > ROGDI expression (F[2,229] = 0.5346, *P* = 0.5866). In the GABAergic rescue, two-way ANOVA detected significant effects of *Rogdi* mutation (F[1,188] = 149.3, *P* < 0.0001) and *GAD1* > ROGDI expression (F[2,188] = 62.12, *P* < 0.0001) as well as their significant interaction (F[2,188] = 9.253, *P* = 0.0001). n.s., not significant, **P < 0.01 to both heterozygous controls in the same genetic backgrounds as determined by Tukey post hoc test. (**c**) Confocal imaging of *Rogdi*-expressing neurons in a whole-mount brain. Fluorescent signals from nuclear green fluorescent proteins expressed by an enhancer-trapping transgene in the *Rogdi* locus (*Rogdi*-Gal4, green) and GABA (magenta) were visualized in the anterior part of the adult fly brain. Bottom panels indicate fluorescence-specific magnifications of the area in the dotted white box. Arrows indicate GABAergic neurons that express *Rogdi*-Gal4. (**d**,**e**) An agonist of GABA_B_-R (SKF-97541) but not GABA_A_-R (THIP) suppresses short sleep caused by constitutive excitation of TH-expressing dopaminergic neurons (*TH* > NaChBac). Data represent average ± SEM (n = 25–50). Two-way ANOVA detected significant interaction of genotypes with the oral administration of SKF-97541 (F[2,225] = 22.67, *P* < 0.0001) but not THIP (F[2,221] = 1.485, *P* = 0.2288). ***P < 0.001 to both heterozygous controls in the same genetic backgrounds; ^#^ *P* < 0.05 to *TH*-Gal4/ + control only as determined by Tukey post hoc test.

We next asked if *Rogdi* expression in either dopaminergic or GABAergic neurons is sufficient to sustain sleep behaviors. Transgenic ROGDI expression in TH-expressing dopaminergic neurons failed to rescue the short sleep phenotypes in *Rogdi* mutants (Fig. 6a, Supplementary Fig. 8a). However, ROGDI expression in GABAergic neurons, driven by the glutamate decarboxylase 1 (*GAD1*)-Gal4 transgene^42^, partially but significantly rescued sleep behaviors in *Rogdi* mutants (Fig. 6b, Supplementary Fig. 8b). To confirm that *Rogdi* is endogenously expressed in GABAergic neurons, we examined *Rogdi*-expressing neurons in adult fly brains. Since our anti-ROGDI antibodies were limited to the detection of endogenous ROGDI proteins in immunoblots, we employed an enhancer trap line that expresses a transgenic Gal4 driver from the *Rogdi* locus (*Rogdi*-Gal4), likely reflecting the endogenous expression of ROGDI. *Rogdi*-Gal4 was broadly expressed in both anterior and posterior regions in the whole-mount brain when its spatial expression was indirectly visualized by green fluorescent proteins (Fig. 6c, Supplementary Fig. 9). Co-immunostaining with anti-GABA antibodies further revealed GABA-positive neurons among other *Rogdi*-expressing neurons, particularly in the anterior brain.

Given that *Rogdi* effects on sleep were suppressed by pharmacological and genetic blockade of DA transmission, we reasoned that the wake-promoting DA pathway may act downstream of the *Rogdi*-expressing GABAergic neurons via metabotropic GABA receptors. To validate this hypothesis, we first examined if RNA interference-mediated depletion of metabotropic GABA receptors in TH-expressing dopaminergic neurons could phenocopy *Rogdi* mutation. However, none of the RNAi lines caused short sleep phenotypes comparable to those in *Rogdi* mutants (Supplementary Fig. 10). While lack of RNAi phenotypes does not necessarily verify the absence of the sleep-promoting metabotropic GABA receptors in dopaminergic neurons, we could not rule out the possibility that the inhibitory GABAergic input to DA pathway might be mediated indirectly by interneurons. To further validate our original hypothesis above, we examined if GABA receptor agonists could suppress short sleep phenotypes caused by the genetic excitation of TH-expressing dopaminergic neurons. Transgenic expression of the bacterial sodium channel NaChBac^43^ in TH-expressing neurons (*TH* > NaChBac) potently shortened daily amount of sleep (Fig. 6d,e) although phenotypic difference between *Rogdi* mutation and constitutive excitation of TH neurons was observed in sleep latency (Supplementary Fig. 11). Nonetheless, the short sleep phenotype was partially but significantly rescued by oral administration of SKF-97541 whereas sleep-promoting effects of THIP were comparable between *TH* > NaChBac flies and their heterozygous controls. These data suggest that GABAergic transmission via metabotropic GABA receptors could titrate the wake-promoting effects of the DA pathway regardless of their dopaminergic expression as consistent with our model for *Rogdi*-dependent sleep regulation.

### Insomnia-like Behaviors in *Rogdi* Mutants Are Sensitized to Select Anti-epileptic Drugs

GABA has long been implicated in many aspects of neural dysfunction including seizures^10–12^, a well-penetrated neurological phenotype in KTS patients. Moreover, sleep deficits frequently accompany epilepsy^13–16^, and their severity is positively correlated in human epileptic patients and animal models^44–46^. In fact, sleep deprivation can cause or even aggravate epileptic seizures^46–48^. Not surprisingly, many anti-epileptic drugs (AEDs), including those promoting GABAergic transmission, also modulate sleep behaviors^15^. We therefore asked if GABA-relevant AEDs could restore sleep behaviors in *Rogdi* mutants.

**Figure 7:**
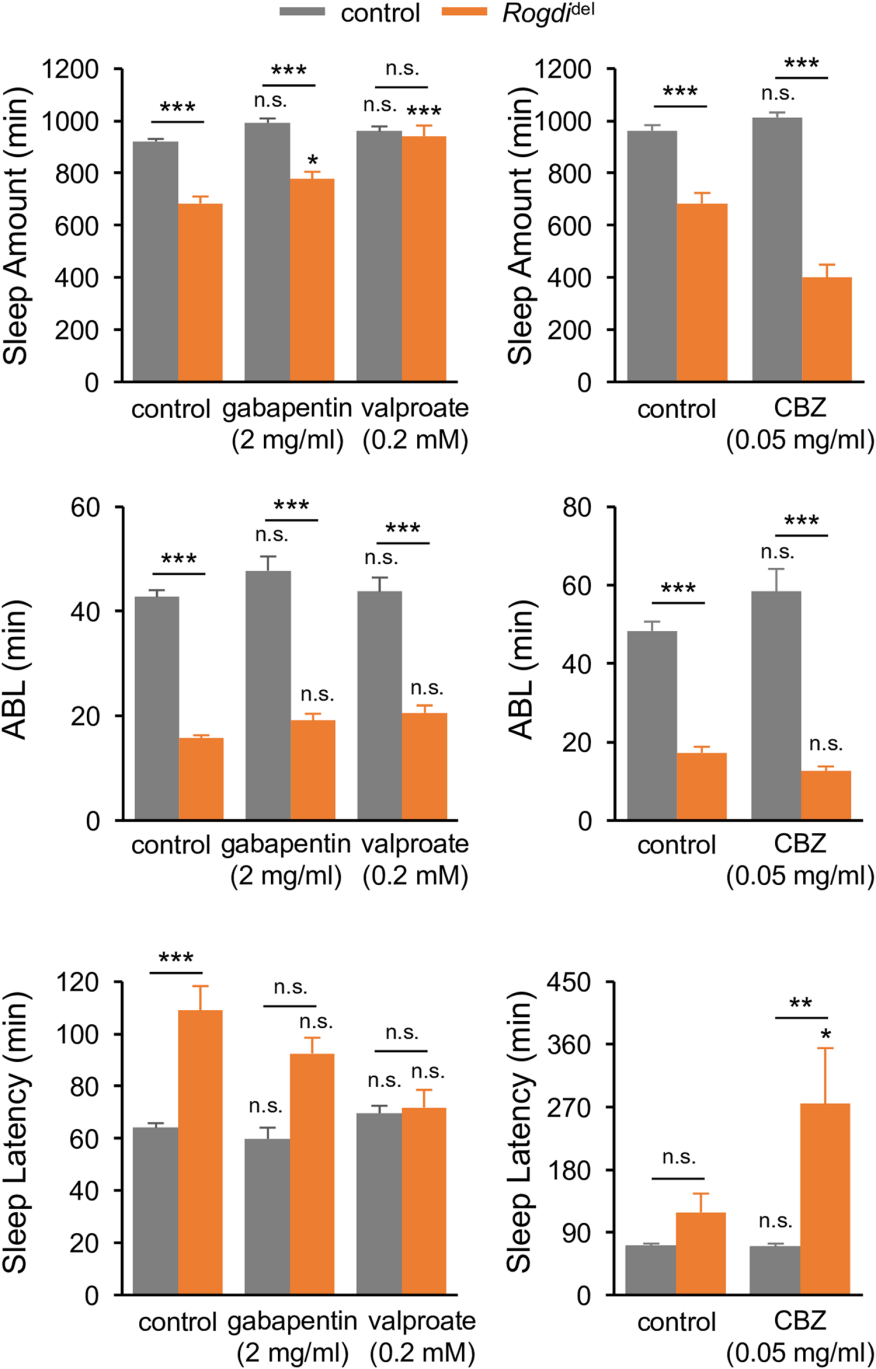
Insomnia-like Behaviors in Rogdi Mutants Are Sensitized to Select Anti-epileptic Drugs. Oral administration of anti-epileptic drugs showed differential effects on short sleep phenotypes in *Rogdi* mutants. Sleep behaviors in individual flies were analyzed similarly to the data presented in Fig. 1. Gray and orange bars indicate wild-type (*w* ^*1118*^) and *Rogdi* ^del^ mutant backgrounds, respectively. Data represent average ± SEM (n = 21–97). Two-way ANOVA detected significant interaction of *Rogdi* mutation with valproate and CBZ on sleep amount (F[1,185] = 17.73, *P* < 0.0001 for valproate; F[1,114] = 24.88, *P* < 0.0001 for CBZ) and sleep latency (F[1,185] = 3.892, *P* = 0.0500 for valproate; F[1,114] = 4.482, *P* = 0.0364 for CBZ). No significant interactions were observed between *Rogdi* mutation and gabapentin. n.s., not significant, *P < 0.05, ***P < 0.001 to no-drug controls in the same genetic backgrounds as determined by Tukey post hoc test.

Gabapentin and valproate are two AEDs that enhance GABAergic transmission and have been shown to ameliorate seizure susceptibility in *Drosophila* ^47, 49, 50^. We found that oral administration of valproate but not gabapentin fully rescued *Rogdi* mutant phenotypes in total sleep amount and sleep latency at a dosage showing negligible effects in control flies (Fig. 7). In contrast, the short sleep phenotypes in *Rogdi* mutants were aggravated by anticonvulsant carbamazepine (CBZ). A previous study demonstrated that CBZ rather suppresses sleep behaviors in *Drosophila* and its wake-promoting effects are, in part, attributable to increased sleep latency by the desensitization of *Resistant to dieldrin*, an ionotropic GABA receptor^51^. We note, however, that these AEDs could modulate neural activities in a GABA-independent manner. For instance, CBZ is known as a blocker of voltage-gated sodium channels^52^ whereas mechanisms underlying anticonvulsant effects of gabapentin and valproate still remain elusive. Nonetheless, these data indicate that *Rogdi* mutant sleep is sensitized to select but not all AEDs relevant to GABAergic transmission and possibly correlate with the observation that seizures in *Rogdi*-associated KTS patients are often resistant to anti-epileptic drugs^53^.

## Discussion

Modeling of neurological diseases and disease-relevant genes has greatly advanced our understanding of the fundamental principles that underlie disease pathogenesis as well as brain function. Here, we established the first genetic model of the KTS-associated disease gene *Rogdi* to demonstrate that *Rogdi* functions as a novel sleep-promoting factor in GABAergic neurons by promoting GABA transmission. While GABA-dependent sleep regulation via ionotropic GABA receptors have been well documented in *Drosophila* ^41, 51, 54–56^, our data suggest that GABAergic transmission via metabotropic GABA receptors might be primarily compromised by *Rogdi* mutation. Furthermore, we identified the wake-promoting DA pathway as a neural locus downstream of *Rogdi*-dependent GABA signaling given that *Rogdi* mutant sleep could be rescued by pharmacological or genetic manipulation of dopaminergic transmission. This sleep-regulatory pathway was further supported by our observation that wake-promoting effects of TH-expressing dopaminergic neurons could be selectively titrated by an agonist of metabotropic GABA receptors. *Rogdi* thus defines a novel pathway coupling these two neurotransmitters to promote baseline sleep in *Drosophila* as exemplified in other behavioral paradigms across species^57–59^. On the other hand, *Rogdi*-dependent GABA transmission might have inhibitory effects on a sleep-promoting neural pathway for sleep homeostasis^60, 61^ to suppress recovery sleep after sleep loss.

What is the molecular basis by which *Rogdi* supports GABAergic transmission and promotes sleep? A possible role of ROGDI as a transcription factor has been suggested by the nuclear localization of human ROGDI protein, particularly in the nuclear envelope of blood mononuclear cells and dermal fibroblasts^2^, and by the conservation of a putatively dimerizing leucine zipper (ZIP) motif among ROGDI homologs^5^. Several lines of evidence, however, argue against this possibility. bZIP transcription factors possess basic residues followed by their ZIP domains whereas ROGDI protein lacks the canonical motif (i.e., basic residues) for DNA-binding activity and nuclear localization. *Drosophila* ROGDI actually displays its subcellular distribution in both nucleus and cytoplasm of cultured cells or adult fly neurons (Supplementary Fig. 3) although the exclusive nuclear localization might not be a prerequisite for transcriptional activities. We recently reported the crystal structure of human ROGDI protein^62^ and showed that, unlike other bZIP transcription factors, human ROGDI protein exists as a monomer containing two structurally distinguishable domains (designated as α and β domains, respectively) (Supplementary Fig. 2a). The α domain exhibits an α-helical bundle that consists of H1, H2, H3, and H6 helices. In fact, the ZIP-like motif in the α domain appears to mediate their intramolecular interactions, contributing to the overall structure and stability of a monomeric ROGDI protein. Based on sequence homology between *Drosophila* and human ROGDI proteins, we predict that *Rogdi*[del] allele removes the majority of the first helix including the repeated leucine residues in the α domain and the first three strands in the β domain, explaining the instability of ROGDI^del^ proteins (Fig. 1c, Supplementary Fig. 2b). A smaller but comparable deletion of the ZIP-like motif has been reported in a KTS patient with a splicing mutation in human *Rogdi* gene^5^. In addition, a functional study in cervical cancer cell lines demonstrated *Rogdi* effects on cell cycle progression and radio-sensitivity^9^. However, further investigations will be required to understand how these cellular phenotypes could be linked to the molecular and neural function of ROGDI protein.

What will be the relevance of our findings to KTS pathogenesis? Genetic heterogeneity has been reported among KTS patients, indicating that *Rogdi*-independent genetic mutations could contribute to KTS pathogenesis^4, 63^. A recent study indeed showed that familial mutations in a sodium-citrate transporter gene *SLC13A5* are the second genetic cause of KTS^64^. The pathogenic phenotypes commonly found in *Rogdi*- and *SLC13A5*-associated KTS gives rise to the intriguing possibility that these two genes might work together to control the intracellular levels of citrate^65^. This idea is further supported by the relevance of citrate metabolism to neurological phenotypes in KTS patients. Neurons are energetically dependent on astrocytes because neurons lack pyruvate carboxylase, an enzyme that converts pyruvate to oxaloacetate in the citric acid cycle^66, 67^. *SLC13A5* plays an important role in the transport of glial citrate into neurons to supplement the neuronal citric acid cycle and thereby supply cellular energy^68, 69^. Furthermore, citrate, an intermediate in the citric acid cycle, acts as a precursor of α-ketoglutarate, which can be metabolized to glutamate and GABA^70^, implicating *SLC13A5* in the biogenesis of GABA. Consistently, anti-epileptic drugs that elevate GABAergic transmission rescued the seizure phenotypes in *SLC13A*-associated KTS patients^65^. In addition, we showed that the pharmacological enhancement of GABAergic transmission by oral administration of GABA-T or GAT inhibitors was sufficient to rescue the short sleep phenotypes in *Rogdi* mutant flies.

Our genetic studies strongly implicate *Rogdi* function in GABAergic transmission, providing the first clue to understanding the neurological phenotypes observed in KTS patients. Molecular and neural deficits selectively caused by *Rogdi* mutation might explain why seizures in *Rogdi*-associated KTS are often resistant to anti-epileptic drugs^53^, as exemplified by our pharmacological rescue of *Rogdi* mutant sleep with a specific AED (Fig. 7). Future studies should thus address if *Rogdi* mutant flies display seizure-like behaviors similarly as in KTS patients and if *Rogdi*-dependent neural relay of GABAergic transmission controls seizure susceptibility in parallel with baseline sleep. In addition, it will be important to determine whether sleep deficiencies are also observed in KTS patients and whether reduced GABAergic transmission in *Rogdi*- and, possibly, *SLC13A5*-associated KTS patients is responsible for their neural dysfunctions, including early-onset seizures. Taken together, our genetic model would constitute an important platform for elucidating the molecular and neural pathogenesis underlying KTS and hint towards a precise development of a therapeutic strategy for KTS in the future.

## Materials and Methods

### Fly Stocks

All flies were maintained in standard cornmeal–yeast–agar medium under 12 hour light: 12 hour dark cycles at 25 °C. *w* ^*1118*^ (BL5905), *TH*-Gal4 (BL8848), *TDC2*-Gal4 (BL9313), *GAD1*-Gal4 (BL51630), UAS-NaChBac-EGFP (BL9467), UAS-GABA-B-R1 RNAi (BL28353, BL51817), and UAS-GABA-B-R2 RNAi (BL27699) stocks were obtained from the Bloomington *Drosophila* Stock Center. *dumb* ^2^ (f02676) was obtained from the Exelixis Collection at Harvard Medical School. UAS-TNT was described previously^71^. *Rogdi*-Gal4 (C0113) was a gift from J. Dubnau (Cold Spring Harbor Laboratory). *Rogdi* ^P1^ (12866R-1) was obtained from the National Institute of Genetics, Japan. The imprecise excision of the P element insertion in *Rogdi* ^P1^ mutants was induced by genetic crosses to a transgenic line expressing a transposase. Excision lines were individually established and screened for large deletions in the genomic *Rogdi* locus to isolate the *Rogdi* ^del^ allele. *Rogdi* mutant stocks were isogenized by outcrossing to *w* ^*1118*^ backgrounds more than six times prior to the behavioral analyses. A full-length cDNA encoding ROGDI-PA was PCR-amplified from head cDNA library and inserted into a modified pUAS-C5 with a C-terminal 3xFLAG tag^72^. The UAS-ROGDI-3xFLAG transgene was then injected into *w* ^*1118*^ to establish several independent UAS-ROGDI lines (BestGene Inc).

### Behavioral Analyses

Behavioral data were recorded using the *Drosophila* Activity Monitor system (Trikinetics) under 12-hour light: 12-hour dark cycles at 25 °C. Each male fly was transferred into a 65 × 5 mm glass tube containing 5% sucrose and 2% agar food. Locomotor activity in individual flies was quantified by counting the number of infrared beam crosses per minute. A sleep bout was defined as a behavioral episode during which flies did not show any activity for 5 minutes or longer. Sleep parameters were analyzed with an Excel macro^73^. Mechanical sleep deprivation was conducted by Sleep Nullifying Apparatus (SNAP)^74^ while sleep behaviors in individual flies were continuously monitored on the SNAP device before or after the sleep deprivation. Since *Rogdi* ^P1^ allele has an unrelated RNA interference transgene and induces its overexpression by Gal4 drivers, most behavioral tests including transgenic rescue experiments were performed in *Rogdi* ^del^ mutant backgrounds.

### Drug Treatment

AMPT (Sigma), L-DOPA (Sigma), EOS (Tokyo Chemical Industry), DABA (Sigma), and valproic acid (Sigma) were directly dissolved at the indicated concentrations in the food used during behavioral testing, which contained 5% sucrose and 2% agar (‘behavior food’). THIP (Tocris), SKF-97541 (Tocris), CBZ (Acros), and gabapentin (Sigma) were first dissolved at 10 mg/ml (THIP, SKF-97541, CBZ) or 100 mg/ml (gabapentin) and then diluted in the behavior food. For AMPT treatment, flies were fed on AMPT-containing behavior food for 12 hours in the dark phase then switched to standard behavior food at the beginning of the next day; subsequent sleep behaviors were monitored for 24 hours. The wake-promoting effects of L-DOPA were similarly assessed except that 25 μg/ml of ascorbic acid was also included in the behavior food and sleep behaviors were monitored on the same day during 24-hour administration of L-DOPA. For EOS, CBZ, and DABA treatments, flies were pre-fed on drug-containing behavior food for 3 days (EOS, CBZ) or 1.5 days (DABA) and their sleep behaviors were monitored for 24 hours while continuing to be fed on drug-containing food. For THIP, SKF-97541, gabapentin, and valproate treatments, flies were pre-fed on drug-containing behavior food for 1.5 days and their sleep behaviors were monitored for 3 days (*Rogdi* mutants and their control flies) or for 24 hours (*TH* > NaCh and their control flies) while continuing to be fed on drug-containing food.

### Western Blot Analysis

Two rabbits were immunized with bacterially purified, full-length *Drosophila* ROGDI protein to generate polyclonal anti-ROGDI antibodies (AbFrontier). Fly heads were collected and homogenized in a lysis buffer (25 mM Tris-Cl pH 7.5, 300 mM NaCl, 10% glycerol, 1 mM EDTA, 1 mM dithiothreitol, 0.5% Nonidet P-40, 1 mM phenylmethylsulfonyl fluoride). Protein extracts were resolved by 10% SDS-PAGE, transferred to Protran nitrocellulose membranes (GE Healthcare), and immunoblotted with rabbit anti-ROGDI (diluted at 1:2000) or mouse anti-TUBULIN (diluted at 1:2000, Developmental Studies Hybridoma Bank) antibodies. After overnight incubation with the primary antibodies at 4 °C with gentle agitation, protein blots were further incubated with horseradish peroxidase-conjugated secondary antibodies (diluted at 1:5000, Jackson ImmunoResearch) and detected by Clarity Western ECL blotting substrate (Bio-Rad) using ImageQuant LAS 4000 (GE Healthcare).

### Whole-brain Imaging

Adult fly brains were dissected in phosphate-buffered saline (PBS), fixed in PBS containing 3.7% formaldehyde, and then blocked with 0.5% normal goat serum (NGS) in PBS containing 0.3% Tritox X-100 (PBS-T). Dissected brains were incubated with rabbit anti-GABA antibody (diluted in PBS-T containing 0.5% NGS and 0.05% sodium azide at 1:1000, Sigma) for 2 days at 4 °C. After washing with PBS-T, brains were further incubated with anti-rabbit Alexa Flour 594 antibody (diluted at 1:600, Jackson ImmunoResearch) for 1 day at 4 °C, washed with PBS-T, and then mounted in a VECTASHIELD mounting medium (Vector Laboratories). Confocal images were acquired with a Laser Scanning Confocal Microscope (FV1000, Olympus) and analyzed in ImageJ software.

### Quantification of Protein Stability

*Drosophila* S2 cells were cultured in Shields and Sang M3 insect medium (Sigma-Aldrich) supplemented with 10% fetal bovine serum and 1% penicillin-streptomycin (ThermoFisher Scientific) at 25 °C. Expression vectors for either wild-type ROGDI or ROGDI^del^ protein with a C-terminal 3xFLAG tag were transiently transfected using Effectene Transfection Reagent according to the manufacturer’s instructions (QIAGEN). Transfected cells were further incubated with 100 μg/ml of cycloheximide to block protein synthesis for the indicated hours before their harvest at 48 hours after transfection. Total cell extracts were resolved by SDS-PAGE and immunoblotted with anti-FLAG or anti-TUBULIN antibodies. Intensities from immunoblotting signals were quantified using ImageJ software. Relative levels of ROGDI proteins at each time-point were calculated by normalizing to those of TUBULIN proteins. Fitting curves for the time-dependent decay of ROGDI proteins were generated using Excel and used to estimate their half-life in hours.

### Data Availability

All data analyzed during this study are included in this published article (and its Supplementary Information file). The raw datasets generated during the current study are available from the corresponding authors on reasonable request.

## Electronic supplementary material

Supplementary Figures
