# Supplementary material for: Rogdi Defines GABAergic Control of a Wake-promoting Dopaminergic Pathway to Sustain Sleep in Drosophila
Source: Sci Rep. 2017 Sep 12;7:11368. doi: 10.1038/s41598-017-11941-3 (PMC5595912; doi:10.1038/s41598-017-11941-3)
Supplement: Supplementary file 1 — Supplementary Figures [file 41598_2017_11941_MOESM1_ESM.pdf]

# *Rogdi* Defines GABAergic Control of a Wake-promoting Dopaminergic Pathway to Sustain Sleep in *Drosophila*

Minjong Kim[1]*, Donghoon Jang[2]*, Eunseok Yoo[1]*, Yangkyun Oh[2], Jun Young Sonn[2], Jongbin Lee[2], Yoonhee Ki[1], Hyo Jin Son[4], Onyou Hwang[4], Changwook Lee[1,3], Chunghun Lim[1]**, and Joonho Choe[2]**

[1] School of Life Sciences, Ulsan National Institute of Science and Technology, Ulsan 44919, Republic of Korea
[2] Department of Biological Sciences, Korea Advanced Institute of Science and Technology, Daejeon 34141, Republic of Korea
[3] Cell Logistics Research Center, Gwangju Institute of Science and Technology, Gwangju 61005, Republic of Korea
[4] Department of Biochemistry and Molecular Biology, University of Ulsan College of Medicine, Seoul 05505, Republic of Korea

*co-first authors; **co-corresponding authors
Correspondence and requests for materials should be addressed to C.Lim (email: clim@unist.ac.kr) or J.C. (email: jchoe@kaist.ac.kr)

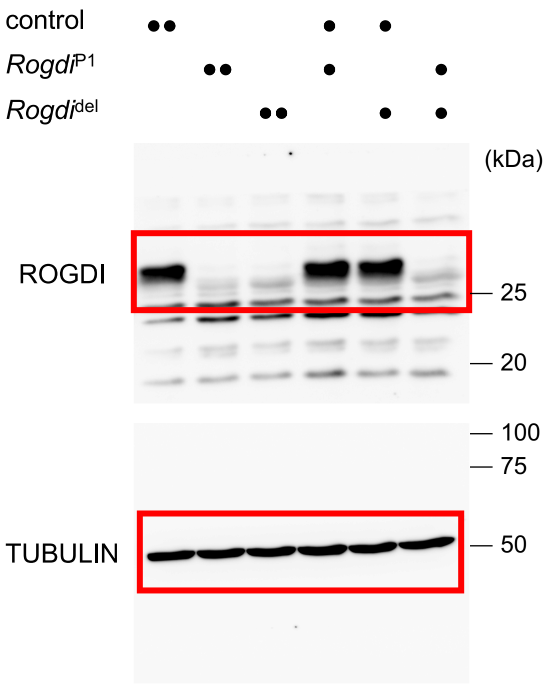


**Supplementary Figure 1. *Rogdi* mutant flies barely express ROGDI proteins.** Head extracts from wild-type and *Rogdi* mutant flies were resolved by SDS-PAGE and immunoblotted with anti-ROGDI (top) and anti-TUBULIN (bottom, loading control) antibodies. Genotypes were shown at the top. Protein size markers were shown on the right. Red rectangles include specific protein bands detected by each antibody in the full-length blot images. The cropped blot images were shown in Fig. 1b.

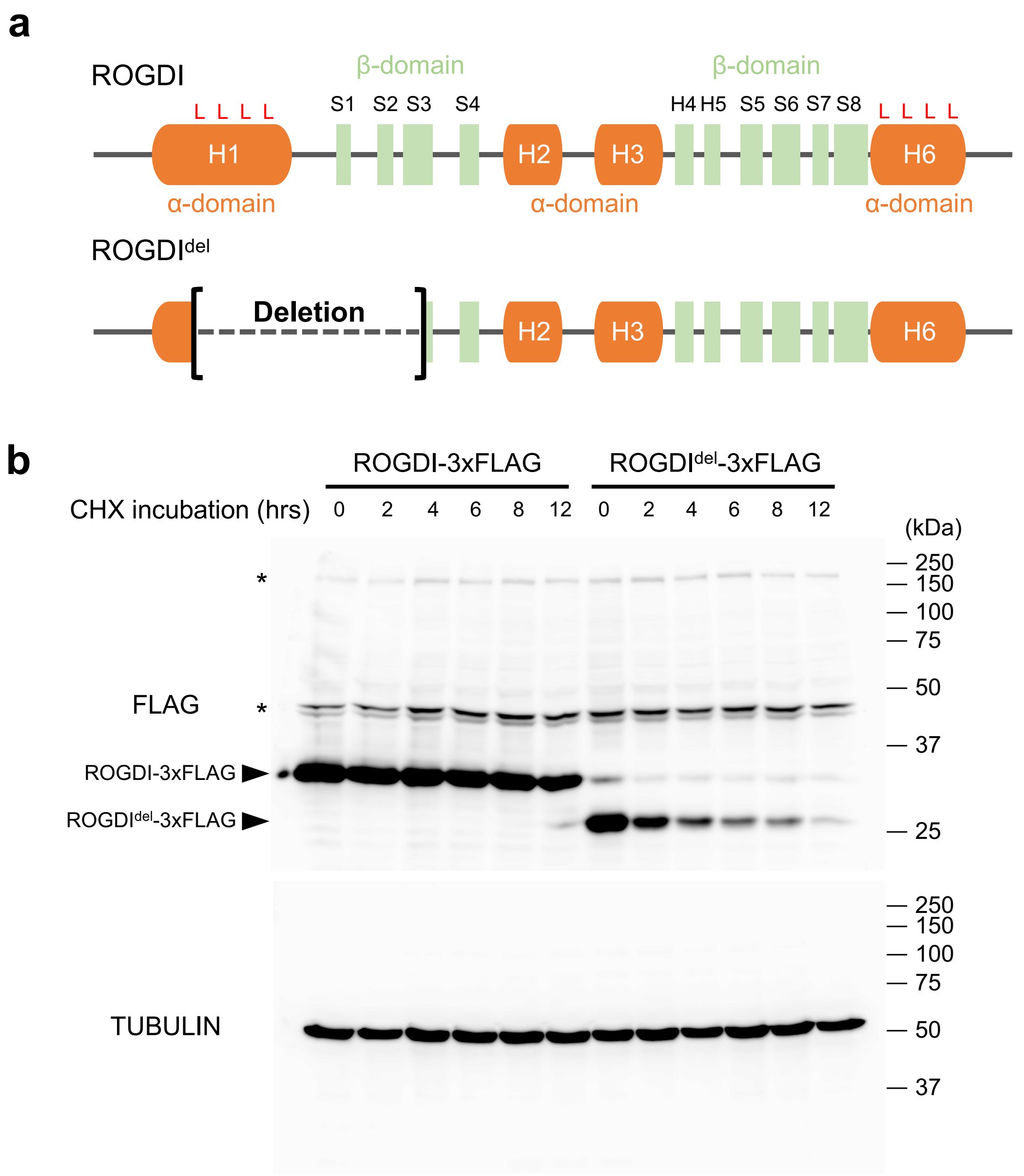


**Supplementary Figure 2. ROGDI$^{del}$ proteins have shorter half-life than wild-type.** (**a**) A schematic of secondary structures and domain organization in wild-type ROGDI (top) and ROGDI$^{del}$ protein (bottom). H and S denote alpha-helix and beta-sheet, respectively. L indicates repetitive leucine residues that constitute leucine zipper-like structure. (**b**) S2 cells were transfected with the expression vector for wild-type ROGDI or ROGDI$^{del}$ proteins with a C-terminal 3xFLAG tag. Where indicated, 100 ug/ml of cycloheximide (CHX) was added to the cell culture media to block protein synthesis before harvest. Total cell extracts were prepared at 48 hours after transfection, resolved by SDS-PAGE, and immunoblotted with anti-FLAG (top) and anti-TUBULIN (bottom, loading control) antibodies. A representative result was shown from three independent experiments. Asterisks indicate non-specific bands with cross-reactivity. Protein size markers were shown on the right.

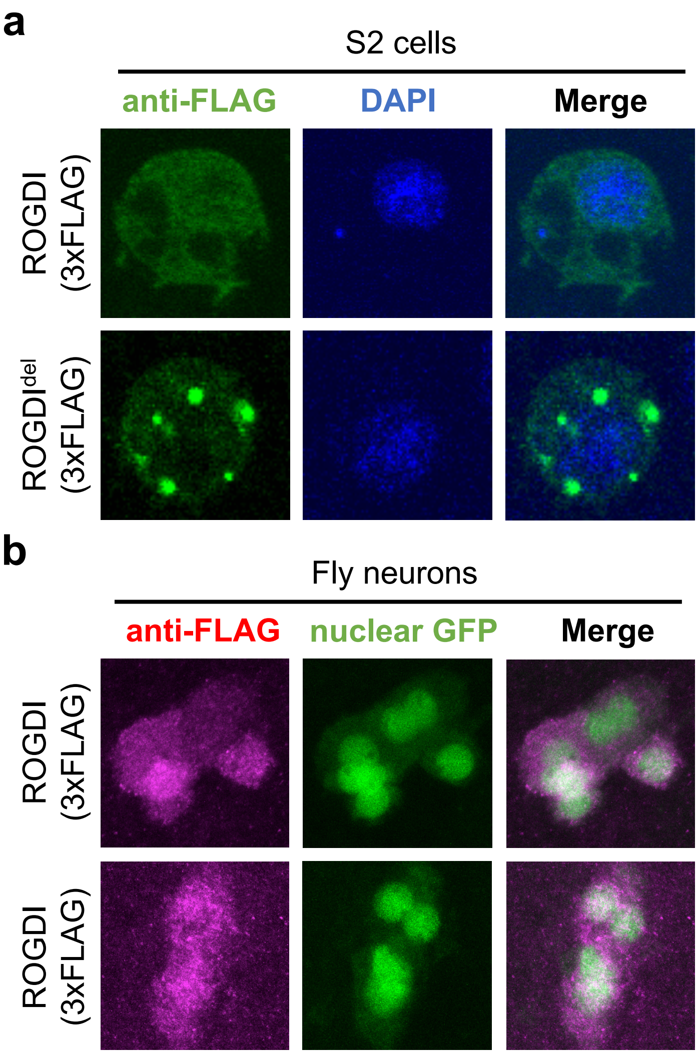


**Supplementary Figure 3. ROGDI$^{del}$ proteins localize to cytoplasmic inclusions.** (**a**) cDNAs corresponding to the coding sequences of wild-type or *Rogdi*$^{del}$ allele fused to a 3xFLAG tag were overexpressed in *Drosophila* S2 cells. The subcellular localization of ROGDI proteins was visualized by immunostaining with anti-FLAG antibody (green) while nuclei were co-stained with 4′,6-Diamidino-2-phenylindole dihydrochloride (DAPI, blue). (**b**) Wild-type ROGDI proteins with the 3xFLAG tag (magenta) were expressed in circadian pacemaker neurons of adult fly brain by *Pigment-dispersing factor* (*Pdf*)-Gal4 driver. Nuclear green fluorescent proteins (GFP) were co-expressed to visualize the nuclei in PDF-expressing neurons.

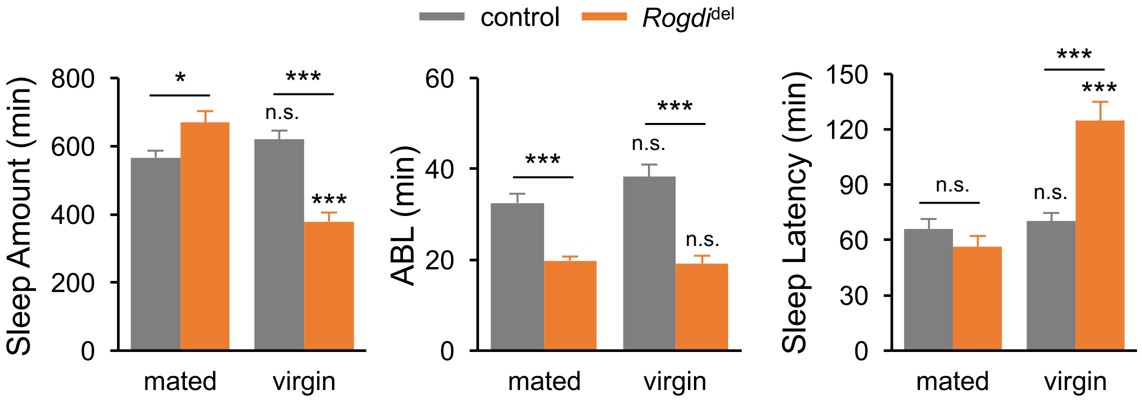


**Supplementary Figure 4. Wake-promoting effects of *Rogdi* mutation are gated by mating status in female flies.** Sleep behaviors in individual female flies were analyzed as similarly in Fig. 1. Gray and orange bars indicate wild-type ($w^{1118}$) and *Rogdi*$^{del}$ mutant backgrounds, respectively. Data represent average ± SEM (n=37–47). Two-way ANOVA detected significant interactions between *Rogdi* mutation and mating status on sleep amount (F[1,161] = 40.79, $P$ < 0.0001) and sleep latency (F[1,161] = 23.78, $P$ < 0.0001) but not on average sleep bout length (ABL) (F[1,161] = 2.559, $P$ = 0.1116). In addition, significant effects of *Rogdi* mutation (F[1,161] = 62.31, $P$ < 0.0001) but not mating status (F[1,161] = 1.597, $P$ = 0.2081) were found on ABL. n.s., not significant, ***$P$ < 0.001 to mated female flies in the same genetic backgrounds as determined by Tukey post hoc test.

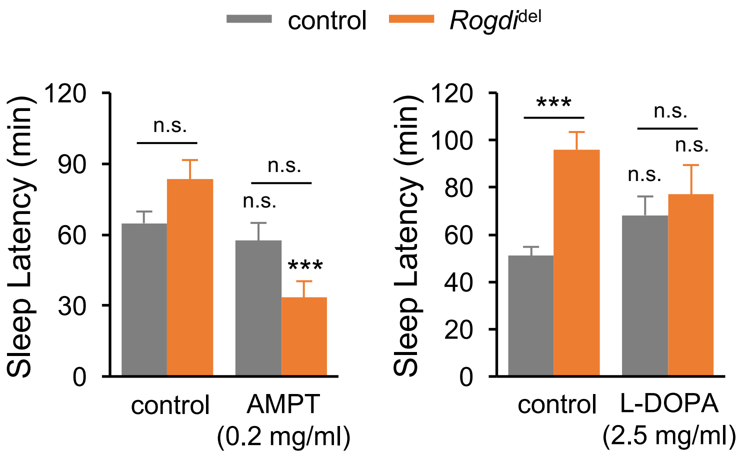


**Supplementary Figure 5. Oral administration of AMPT or L-DOPA affects sleep latency differentially in wild-type and *Rogdi* mutants.** Sleep behaviors in individual flies were analyzed as similarly in Fig. 1. Gray and orange bars indicate wild-type ($w^{1118}$) and $Rogdi^{del}$ mutant backgrounds, respectively. Data represent average ± SEM (n=30–63). Two-way ANOVA detected significant interaction of *Rogdi* mutation with AMPT ($F[1,155] = 9.64$, $P = 0.0023$) and L-DOPA ($F[1,185] = 5.215$, $P = 0.0235$). n.s., not significant, ****P* < 0.001 to no-drug controls in the same genetic backgrounds as determined by Tukey post hoc test. AMPT, alpha-methyl-p-tyrosine (an inhibitor of tyrosine hydroxylase); L-DOPA, L-3,4-dihydroxyphenylalanine (a precursor of DA).

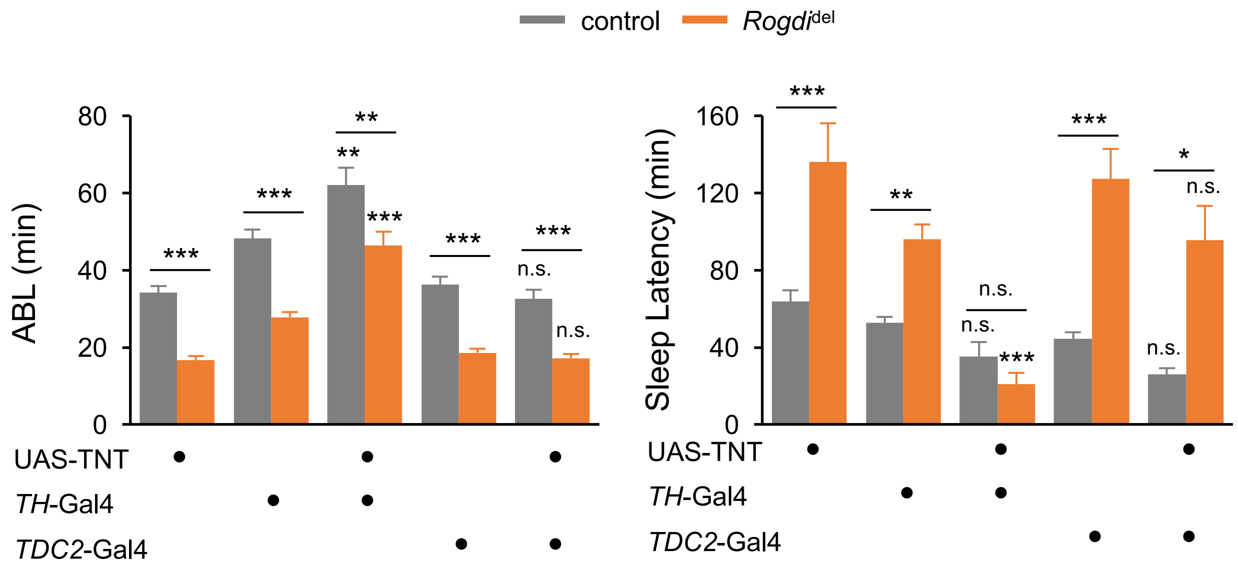


**Supplementary Figure 6. Blocking synaptic transmission in dopaminergic (*TH*>TNT, tetanus toxin light chain) but not octopaminergic (*TDC2*>TNT) neurons masks wake-promoting effects of *Rogdi* mutation.** Sleep behaviors in individual flies were analyzed as similarly in Fig. 1. Gray and orange bars indicate wild-type ($w^{1118}$) and *Rogdi*$^{del}$ mutant backgrounds, respectively. Data represent average ± SEM (n=23–80). Two-way ANOVA detected significant interaction between *Rogdi* mutation and *TH*>TNT on sleep latency (F[2,230] = 6.867, *P* = 0.0013) but not on average sleep bout length (ABL) (F[2,230] = 0.5595, *P* = 0.5723). No significant interactions were detected between *Rogdi* mutation and *TDC2*>TNT (F[2,191] = 0.334, *P* = 0.7165 for ABL; F[2,191] = 0.1111, *P* = 0.8949 for sleep latency). n.s., not significant, **$P$ < 0.01, ***$P$ < 0.001 to heterozygous controls in the same genetic backgrounds as determined by Tukey post hoc test.

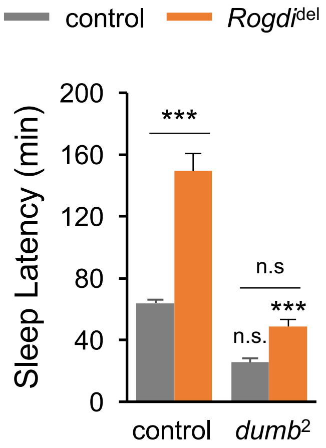


**Supplementary Figure 7. Hypomorphic mutation in a dopamine receptor gene *dumb* suppresses short sleep phenotypes in *Rogdi* mutants.** Sleep behaviors in individual flies were analyzed as similarly in Fig. 1. Gray and orange bars indicate wild-type ($w^{1118}$) and *Rogdi*$^{del}$ mutant backgrounds, respectively. Data represent average ± SEM (n=30–126). Two way ANOVA detected significant effects of *Rogdi* (F[1,280] = 11.07, *P* = 0.001) or *dumb* mutations (F[1,280] = 18.12, *P* < 0.0001) but failed to detect their genetic interaction (F[1,280] = 3.617, *P* = 0.0582). n.s., not significant, ****P* < 0.001 to controls in the same genetic backgrounds as determined by Tukey post hoc test.

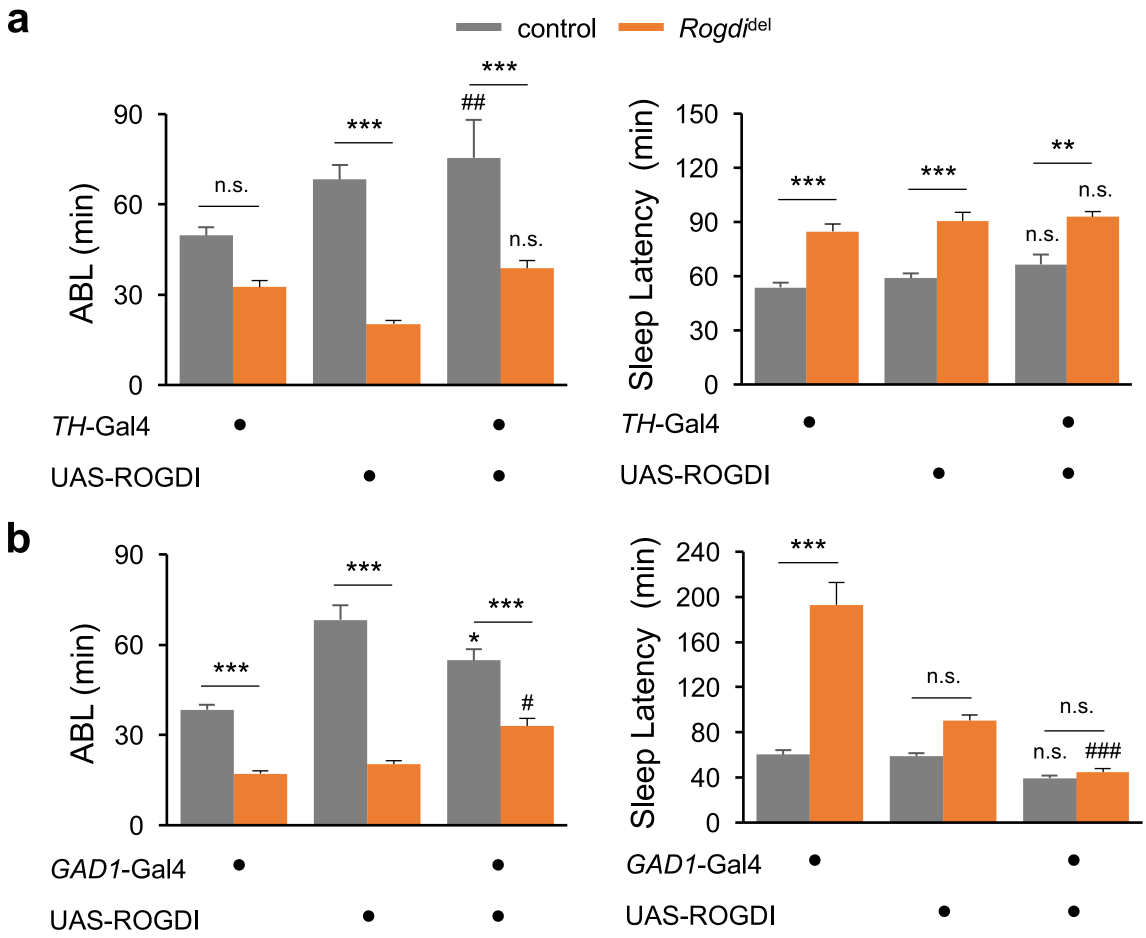


**Supplementary Figure 8. Effects of transgenic ROGDI expression in either dopaminergic or GABAergic neurons on *Rogdi* mutant sleep.** Sleep behaviors in individual flies were analyzed as similarly in Fig. 1. Gray and orange bars indicate wild-type (*w¹¹¹⁸*) and *Rogdi*$^{del}$ mutant backgrounds, respectively. Data represent average ± SEM (n=24–43). (**a**) Two-way ANOVA detected significant interaction of *Rogdi* mutation with *TH*>ROGDI expression on average sleep bout length (ABL) ($F[2,229] = 6.813$, $P = 0.0013$) but not on sleep latency ($F[2,229] = 0.1764$, $P = 0.8384$). (**b**) Two-way ANOVA detected significant interaction between *Rogdi* mutation and *GAD1*>ROGDI expression on both ABL ($F[2,188] = 4.939$, $P = 0.0081$) and sleep latency ($F[2,188] = 12.58$, $P < 0.0001$). n.s., not significant, $^{*}P < 0.05$ to both heterozygous controls; $^{\#}P < 0.05$, $^{\#\#}P < 0.01$, $^{\#\#\#}P < 0.001$ to Gal4/+ controls only as determined by Tukey post hoc test.

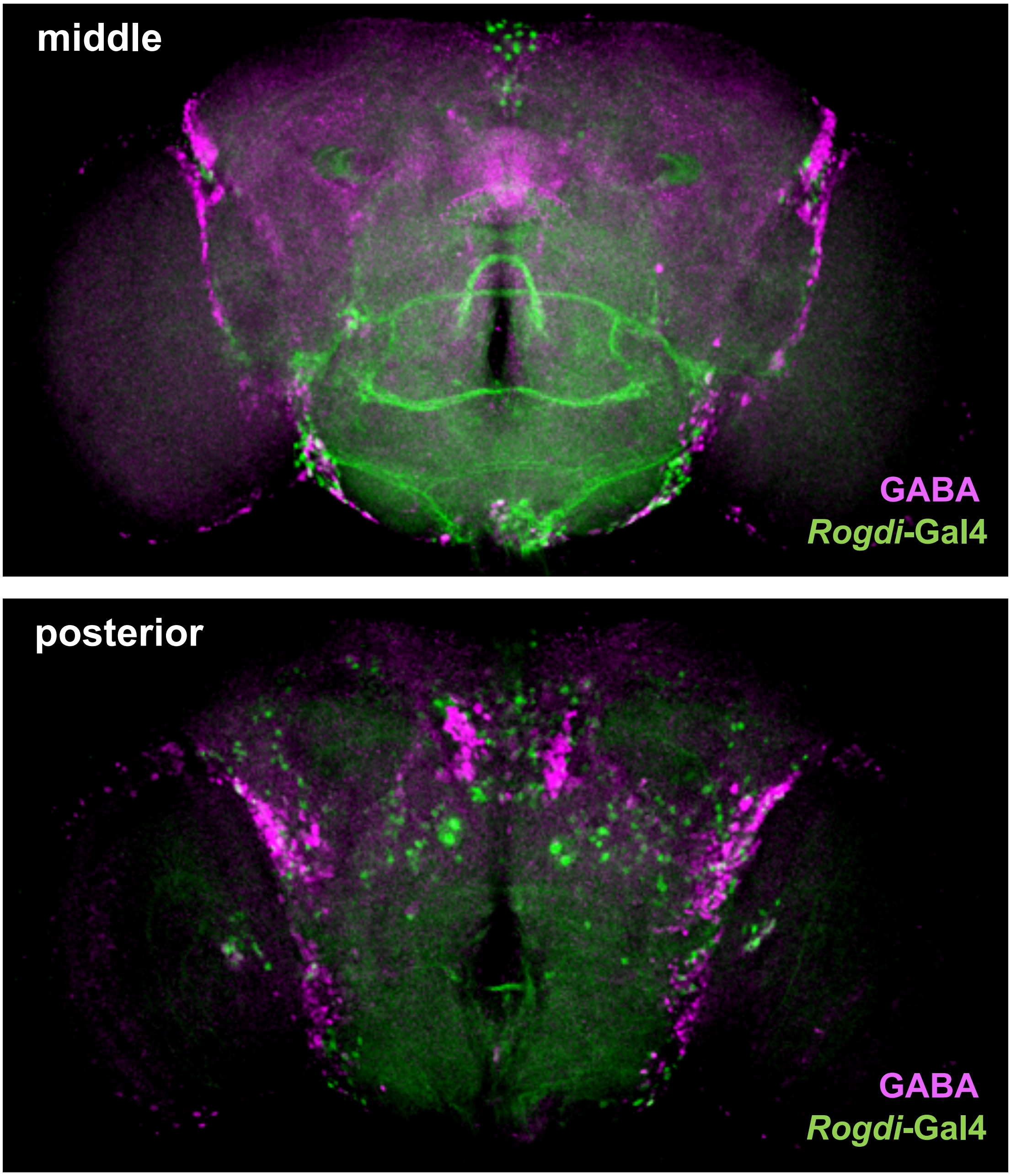


**Supplementary Figure 9. An enhancer trap in *Rogdi* locus displays broad expression in adult fly brain including those neurons positive for anti-GABA staining.** Confocal imaging of *Rogdi*-expressing neurons in a whole-mount brain. Fluorescent signals from nuclear green fluorescent proteins expressed by an enhancer-trapping transgene in the *Rogdi* locus (*Rogdi*-Gal4, green) and GABA (magenta) were visualized from a middle (top) or posterior (bottom) part of adult fly brain.

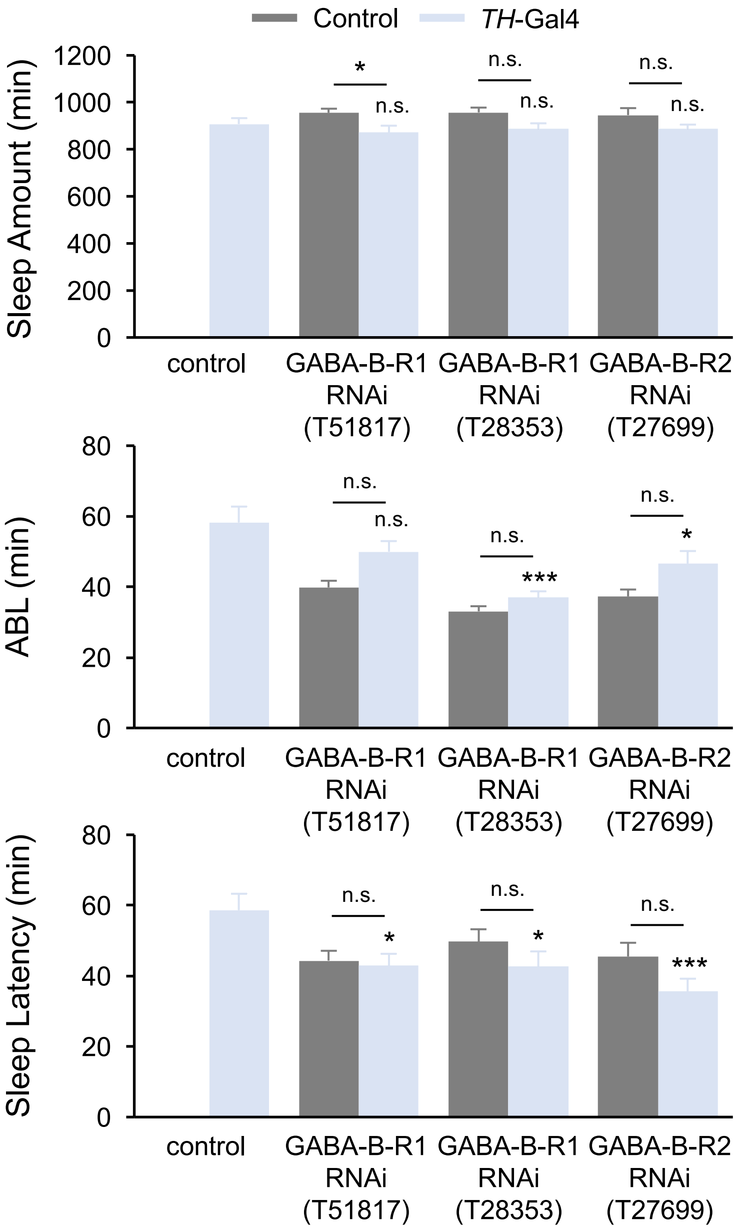


**Supplementary Figure 10. RNA interference-mediated depletion of metabotropic GABA receptors in TH-expressing dopaminergic neurons does not lead to short sleep behaviors.** Each RNA interference (RNAi) transgene was overexpressed in dopaminergic neurons by *TH*-Gal4 driver. Sleep behaviors in individual flies were analyzed similarly to the data presented in Fig. 1. Data represent average ± SEM (n=29–31). n.s., not significant, *$P < 0.05$, ***$P < 0.001$ to *TH*-Gal4 control as determined by one-way ANOVA, Dunnett post hoc test.

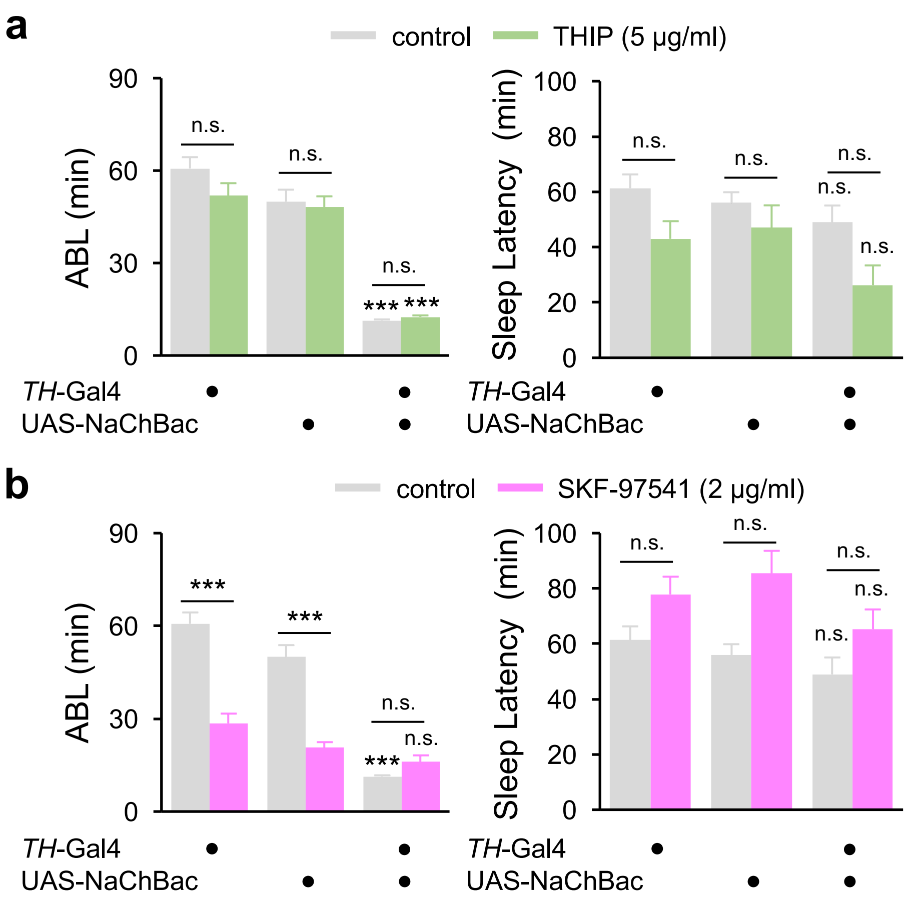


**Supplementary Figure 11. Effects of GABA receptor agonists on short sleep phenotypes caused by genetic excitation of TH-expressing dopaminergic neurons.** (**a**,**b**) TH-expressing dopaminergic neurons were genetically excited by transgenic expression of bacterial sodium channel NaChBac. Sleep behaviors in individual flies were analyzed similarly to the data presented in Fig. 1. Where indicated, THIP or SKF-97541 was fed on transgenic flies to test their effects on ABL and sleep latency. Data represent average ± SEM (n=25–50). Two-way ANOVA detected significant interaction of genotypes with SKF-97541 ($F[2,225] = 20.26$, $P < 0.0001$) but not with THIP ($F[2,221] = 1.162$, $P = 0.3149$) on ABL. No significant interactions between genotypes and GABA receptor agonists were detected on sleep latency ($F[2,221] = 0.6369$, $P = 0.5299$ for THIP; $F[2,225] = 0.3713$, $P = 0.6902$ for SKF-97541). n.s., not significant, ***$P < 0.001$ to both heterozygous controls as determined by Tukey post hoc test.
